# Peripheral transcriptional responses to experimental SARS-CoV-2 inoculation in North American elk cows and calves

**DOI:** 10.1186/s12864-025-11956-5

**Published:** 2025-08-28

**Authors:** Bruna Petry, Kaitlyn M. Sarlo Davila, Alexandra C. Buckley, Eric D. Cassmann, Steven. C. Olsen, Paola M. Boggiatto, Mitchell V. Palmer, Ellie J. Putz

**Affiliations:** 1 Infectious Bacterial Diseases Research Unit, National Animal Disease Center (NADC-USDA), 1920 Dayton Ave, Ames, IA 50010 USA; 2 Oak Ridge Institute for Science and Education, 1299 Bethel Valley Road, Oak Ridge, TN 37830 USA; 3 Ruminant Diseases and Immunology Research Unit, National Animal Disease Center (NADC-USDA), 1920 Dayton Ave, Ames, IA 50010 USA; 4 Virus and Prion Research Unit, National Animal Disease Center (NADC- USDA), 1920 Dayton Ave, Ames, IA 50010 USA; 5 Department of Veterinary Pathology, College of Veterinary Medicine, Iowa State University, Patterson Hall, 1800 Christensen Drive, Ames, IA 50011 USA

**Keywords:** SARS-CoV-2, COVID-19, Coronavirus, RNA-seq, Zoonotic, Elk, *Cervus elaphus canadensis*

## Abstract

**Background:**

Severe acute respiratory syndrome coronavirus 2 (SARS-CoV-2) remains a health risk for humans and other domestic and wildlife species. Recently, North American elk have been identified as seropositive for SARS-CoV-2, thus posing a potential threat to humans and other mammals. In this work, we characterized the peripheral transcriptomic response to experimental SARS-CoV-2 infection in calves and adult elk at different time points.

**Results:**

Significantly differentially expressed genes were identified at 2-, 5-, and 14-days post inoculation (pi) for both age groups. Adult elk presented the greatest number of differentially expressed (DE) genes at all time points, including many genes associated with viral response, immune activation, antibody production, as well as genes associated with coronavirus disease (COVID-19), and coronavirus GO terms and KEGG pathways. Calves presented DE genes associated with viral responses at 5 days pi as well as neurodegenerative-associated genes at 14 days pi. Both adults and calves showed predicted activation of the *ISGF3* and *IFN type I* pathways at day 2 pi and, globally, increased activity related to the coronavirus pathway disease at 5 and 14 days pi.

**Conclusions:**

Collectively, this work provides valuable data characterizing the cervid immune response of elk to viral diseases as well as the response of wildlife to SARS-CoV-2 infection.

**Supplementary Information:**

The online version contains supplementary material available at 10.1186/s12864-025-11956-5.

## Background

Severe acute respiratory syndrome coronavirus 2 (SARS-CoV-2), the cause of coronavirus disease 2019 (COVID-19), remains a public health concern and one of the most severe global public health emergencies in history, killing more than 7 million people around the world (World Health Organization - https://data.who.int/dashboards/covid19/deaths) by March 2025.

In addition to its impact on human health, SARS-CoV-2 is also a concern in various domestic, peridomestic, and wild animals, including cats, dogs, mice, rats, American mink (*Neovison vison*), Eurasian river otters (*Lutra lutra*), ferrets (*Mustela furo*), Syrian hamsters (*Mesocricetus auratus*), gorillas, lions, tigers, Virginia opossums (*Didelphis virginiana*), raccoons (*Procyon lotor*), groundhog (*Marmota monax*), red bats (*Lasiurus borealis*), white-tailed deer (*Odocoileus virginianus*), red deer (*Cervus elaphus*), and fallow deer (*Dama dama*) [1, 2]. At the molecular level, the most defining structural protein of SARS-CoV-2 is the spike (S) protein. During infection, the S protein binds to surface angiotensin-converting enzyme 2 (ACE2), the receptor responsible for virus entrance into the cell, facilitating virus replication [3, 4]. The variability in the specificity of the interaction between viruses and receptors is believed to reflect the range of susceptible hosts. The ACE2 protein of humans has a high degree of homology with that of white-tailed deer, reindeer (*Rangifer tarandus*), and Pierre Davids’s deer (*Elaphurus davidianus*) [5]. Interspecies comparisons of the ACE2 residues from numerous species of Cervidae, show that the sequence of North American elk (*Cervus elaphus canadensis*) is identical to that of Pierre David’s deer and differs only by one amino acid from that of white-tailed deer [5]. White-tailed deer were previously shown to be highly susceptible to infection with viral shedding and transmission to other white-tailed deer [6, 7]. Subsequent surveys of wild white-tailed deer across the United States (US) revealed widespread exposure/infection, which suggests ongoing deer-to-deer transmission and a possible source of deer-to-human infection.

Although white-tailed deer are the most abundant wild ungulate in North America, with approximately 30 million found in the US alone, another closely related but less numerous large ungulate is North American elk. In a recent publication [8] from our laboratory, the susceptibility of calves and adult elk to SARS-CoV-2 infection was investigated. Although elk are moderately permissive to SARS-CoV-2 infection and exhibit seroconversion, viral shedding and tissue distribution are much lower than what is observed in white-tailed deer. Interestingly, viral RNA and viral protein were detectable in lymphoid tissues 21 days after infection in both elk calves and adults. Based on these findings and to further understand virus‒host interactions, we investigated the changes in whole-blood gene expression in elk calves and adults following experimental challenge with SARS-CoV-2.

Understanding the transcriptomic response to infection will continue to shed light on SARS-CoV-2 pathophysiology in mammals, including age-related differences of cervids, which have not been investigated previously. Specifically, this work offers comparative insight into cervid models of infection. While white-tailed deer appear more permissive to infection, the elk transcriptomic profile detailed herein offers a cervid comparative resource valuable to many stakeholders working with ungulates.

## Results

Both cow elk and calf infection were confirmed by PCR, seroconversion, immunohistochemistry, and/or in situ hybridization in all animals, however, no clinical signs of disease were detected throughout the study [8]. We evaluated the RNA-seq whole-blood transcriptomes of calves and adult elk cows experimentally infected with USA-WA1/2020 SARS-CoV-2 on days 0, 2, 5, and 14 post intranasal inoculation.

### Differentially expressed genes between day 0 and days 2, 5, and 14 post-SARS-CoV-2 inoculation in elk calves

**Fig. 1:**
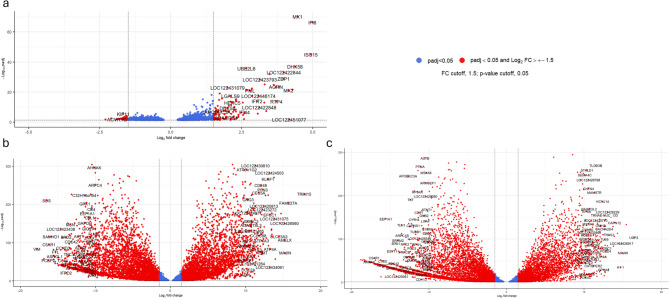
Volcano plot representing differentially expressed (DE) genes with log2-fold changes greater than 1.5 and significant (*p*-adjusted < 0.05) for the calf elk analysis. **a** shows the DE genes on day 2 post inoculation (pi) compared with day 0, (**b**) for day 5 pi, and (**c**) for day 14 pi. Red dots indicate DE genes with an absolute log₂-fold change greater than 1.5 and the dashed horizontal line represents the adjusted *p*-value below 0.05. Blue dots are DE genes with *p*-adjusted < 0.05 and log2-fold change smaller than 1.5.

In elk calves, the comparison of day 2 pi to day 0 revealed 715 genes that were significantly differentially expressed (DE), with 183 downregulated genes and 532 upregulated genes (*p*-adjusted < 0.05) (Fig. 1a, Supplementary material, Table S1). Among the upregulated genes, 24 were involved in the coronavirus disease pathway according to the Kyoto Encyclopedia of Genes and Genomes (KEGG) pathway analysis (Supplementary material, Table S2 and Fig. S1). Interestingly, upregulated genes in this pathway included regulators of inflammation and “cytokine storm” associated immune responses to SARS-CoV-2, such as *CXCL10* (log2fc = 2.60) [9], *IRF3* (interferon regulatory factor 3, log2fc = 0.36), *IRF9* (interferon regulatory factor 9, log2fc = 1.00), *MX1* (MX Dynamin Like GTPase 1, log2fc = 3.70), and *MX2* (MX Dynamin Like GTPase 2, log2fc = 4.70). Both *MX1* and *MX2* are linked to the cellular antiviral immune response [10]. Similarly, *ISG15* (ISG15 ubiquitin-like modifier, log2fc = 3.90) was upregulated and has previously been implicated in inflammatory responses after SARS-CoV-2 exposure [11].

Compared with day 0, the day 5 pi analysis revealed 921 DE genes; 339 downregulated, and 582 upregulated (Fig. 1b, Supplementary material, Table S1). Several upregulated genes were associated with viral immune responses, including the NOD-like receptor signaling pathway (20 genes), herpes simplex virus 1 infection (20 genes), Epstein‒Barr virus infection (19 genes), hepatitis C (17 genes), influenza A (17 genes), human papillomavirus infection (16 genes), and the biological term for defense response to virus (12 genes) (Supplementary material, Table S2 and Fig. S2).

A comparison of day 14 pi versus day 0 revealed the greatest number of DE genes, with a total of 7,179 DE genes, of which 3,602 were downregulated and 3,577 upregulated (Fig. 1c, Supplementary material, Table S1). Some of the upregulated genes were associated with known SARS-CoV-2 players, such as *IRAK1* (interleukin 1 receptor-associated kinase 1, log2fc = 0.87), *IRF3* (interferon regulatory factor 3, log2fc = 0.59), *ISG15* (ISG15 ubiquitin-like modifier, log2fc = 1.30) and *IRF9* (interferon regulatory factor 9, log2fc = 0.66). Additionally, upregulated genes were associated with protein binding, ATP binding, structural constituents of ribosomes, and translation GO terms, as well as thermogenesis and ribosomes as KEGG pathways, all of which are associated with viral responses. Interestingly, several upregulated DE genes were related to neurodegenerative diseases, such as genes related to Alzheimer’s disease: *PSEN2*, *TREM2*, and *GAB2* [12–14]; Parkinson’s disease: *ATP13A2* and *DCTN1* [15]; and amyotrophic lateral sclerosis: *TAF15* and *FUS* [16, 17] (Supplementary material, Table S2 and Fig. S3).

### Differentially expressed genes between day 0 and days 2, 5, and 14 post SARS-CoV-2 inoculation in adult elk

**Fig. 2:**
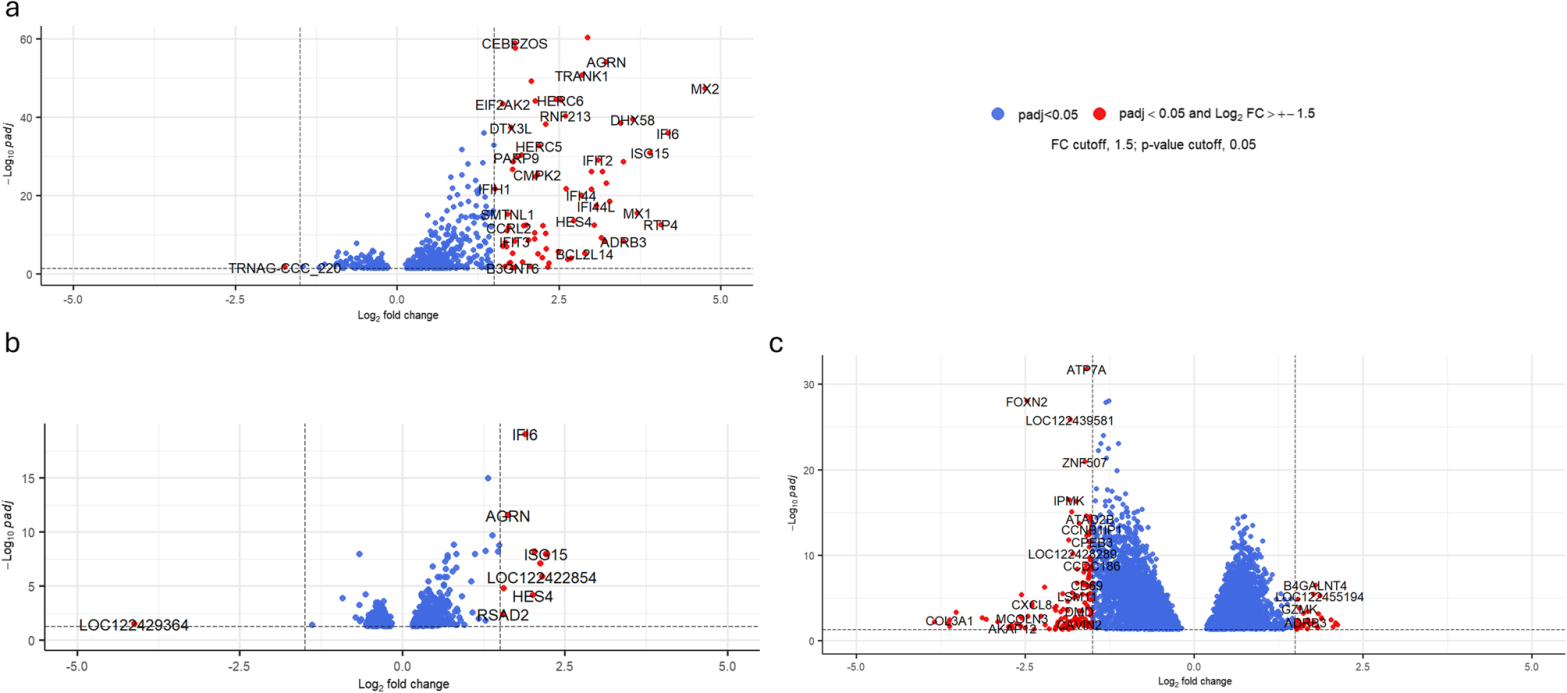
Volcano plot representing DE genes with a log2-fold change greater than 1.5 and significant (*p* adjusted < 0.05) for the DE gene analysis of adult elk. **a** shows the DE genes on day 2 post inoculation (pi) compared with day 0, (**b**) for day 5 pi, and (**c**) for day 14 pi. Red dots indicate DE genes with an absolute log₂-fold change greater than 1.5 and the dashed horizontal line represents the adjusted *p*-value below 0.05. Blue dots are DE genes with *p*-adjusted < 0.05 and log2-fold change smaller than 1.5.

Compared with day 0 samples, the DE genes analysis of adult elk in day 2 pi samples revealed 2,756 DE genes (Supplementary material, Table S3). In total, 1,421 genes were upregulated, whereas 1,234 genes were downregulated (*p* adjusted < 0.05) (Fig. 2a). Upregulated genes were associated with coronavirus disease KEGG pathways, including members of the MAPK family of cascades, such as *MAPK3* (mitogen-activated protein kinase 3) and *MAPKAPK3* (MAPK activated protein kinase 3),with log2-fold change values = 0.70 and 0.47, respectively; *Myd88* (myeloid differentiation primary response gene, log2fc = 0.57); *IRF3* (interferon regulatory factor 3, log2fc = 0.84); *ISG15* (ISG15 ubiquitin-like modifier, log2fc = 4.94); *STAT1* (signal transducer and activator of transcription 1, log2fc = 0.99); and *STAT2* (signal transducer and activator of transcription 2, log2fc = 1.00). Most of these genes are involved in various immune system pathways, including antiviral defense (Supplementary material, Table S4 and Fig. S4).

Analysis revealed 9,708 DE genes in the adult elk between day 5 pi and day 0; of these genes, 5,399 were downregulated, and 4,309 were upregulated (*p*-adjusted < 0.05) (Fig. 2b, Supplementary material, Table S3). Several genes presented large log2-fold changes in both directions (up and downregulated). Examples include *VIM* (vimentin, log2fc = −17.77), *ADA* (adenosine deaminase, log2fc = −16.47), *TRIM15* (tripartite motif containing 15, log2fc = 17.82), *FAM227A* (family with sequence similarity 227 Member A, log2fc = 15.70) and *MAJIN* (membrane anchored junction protein, log2fc = 15.29). Extended results can be found in Supplementary material, Table S4 and Fig. S5. Among the genes upregulated for this comparison, 106 were associated with the coronavirus disease KEGG pathway and included *ACE2* (angiotensin-converting enzyme 2, log2fc = 2.87), *TNF* (tumor necrosis factor, log2fc = 3.46), and MAPK family members, such as *MAPK4* (mitogen-activated protein kinase 4, log2fc = 9.34), *MAPK11* (mitogen-activated protein kinase 11, log2fc = 10.06), and *ISG15* (ISG15 ubiquitin-like modifier, log2fc = 3.21).

Between day 14 pi and day 0, a total of 3,950 genes were DE; 1,799 were downregulated, and 2,151 were upregulated, with a *p*-adjusted < 0.05 (Fig. 2c, see Supplementary material, Table S3). As observed on day 5 pi, there were also large log2-fold changes in *ALAS2* (5’-Aminolevulinate Synthase 2, log2fc = −19.11), *FTH1* (Ferritin Heavy Chain 1, log2fc = −19.03), *GNAI2* (G Protein Subunit Alpha I2, log2fc = −18.78), *UCP3* (Uncoupling Protein 3, log2fc = 16.43), *MAJIN* (Membrane Anchored Junction Protein, log2fc = 15.04) and *H1-1* (H1.1 Linker Histone, Cluster Member, log2fc = 14.69). KEGG pathway analysis revealed 101 upregulated DE genes associated with the coronavirus disease - COVID-19 KEGG pathway, including *MAS1* (MAS1 Proto-Oncogene, G Protein-Coupled Receptor, log2fc = 10.46), *TNF* (Tumor necrosis factor, log2fc = 4.82), MAPK family members such as *MAPK11* (Mitogen-Activated Protein Kinase 11, log2fc = 7.10) and *MAPK12* (Mitogen-Activated Protein Kinase 12, log2fc = 4.69). Genes such as *UCP3* (uncoupling protein 3), *MAJIN* (membrane anchored junction protein), *H1-1* (H1.1 linker histone, cluster member) and *HAS1* (hyaluronan synthase 1) presented log2fc values of 16.43, 15.04, 14.69 and 14.32, respectively. *ISG15* was also upregulated, with log2fc = 1.17 (Supplementary material, Table S4 and Fig. S6).

### Comparison of conserved differentially expressed genes between elk calves and adults

**Fig. 3:**
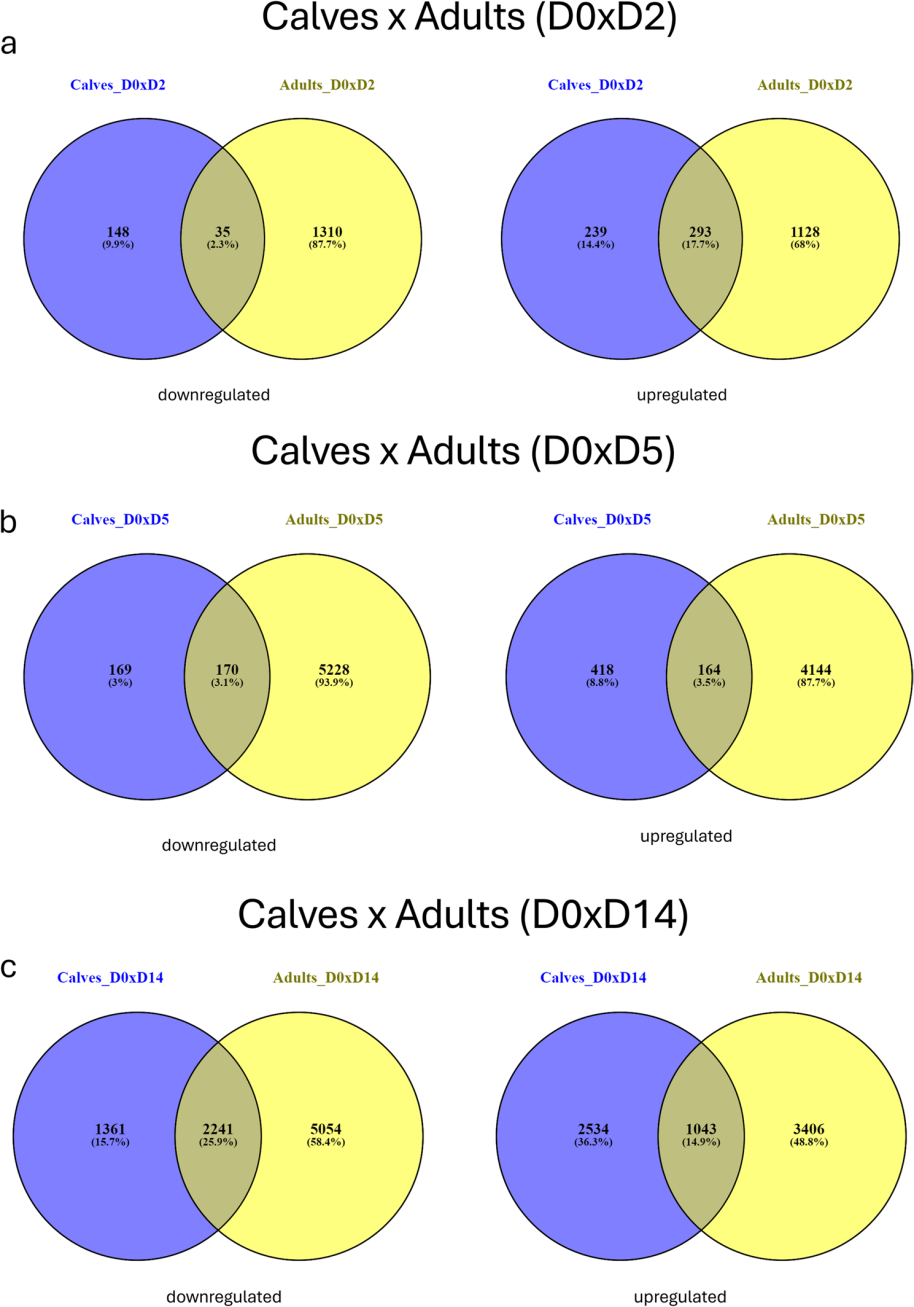
Conserved differentially expressed (DE) genes between calf and adult elk groups. Shown are both significantly downregulated and upregulated genes. **a** depicts DE genes at day 2 post-inoculation (pi), (**b**) 5 days pi. and (**c**) day 14 pi in calves and adult elk.

We also compared the conservation of significantly up- and down-regulated DE genes (Fig. 3), within timepoints between adults and calves to understand the similarities and differences in immune response between elk age groups. When the downregulated genes between the adult and calf groups on day 2 pi vs. day 0 were compared, 35 genes were conserved . Most shared DE genes were associated with the Golgi apparatus, including *STK26* (serine/threonine-protein kinase 26) and *HOOK3* (hook microtubule tethering protein 3) but also include *GEMIN6* (gem nuclear organelle-associated protein 6), a gene previously associated with SARS-CoV-2 infection [18] (Fig. 3a). Among the 239 upregulated genes shared between adults and calves, 19 genes were related with the coronavirus disease-COVID-19 KEGG pathway, and the others were associated with additional antiviral responses, including herpes simplex virus 1 infection, influenza A, the defense response to a virus, hepatitis C, and NOD-like receptor signaling pathways.

When DE genes from both ages of elk were compared between day 5 pi and day 0 (Fig. 3b), 170 downregulated genes were shared. Many of these genes had GO terms related to the nucleus, regulation of transcription by RNA polymerase II, DNA-binding transcription factor activity, RNA polymerase II-specific and ATP binding. The associated KEGG pathways included the regulation of the actin cytoskeleton, cellular senescence and the MAPK signaling pathway. On the upregulated side, 164 genes were shared between adults and calves, 10 of which were associated with the KEGG pathways for coronavirus disease-2019 (COVID-19), other genes related to the NOD-like receptor signaling pathway, and different viral infections, such as influenza A, hepatitis C, Epstein–Barr virus infection, and herpes simplex virus 1 infection.

A comparison of the adult and calf DE gene lists between day 14 pi and day 0 (Fig. 3c) revealed 2,241 downregulated shared genes. The genes with reduced expression were associated with KEGG pathways related to viral infections, including herpes simplex virus 1 disease, human papillomavirus infection, and human cytomegalovirus infection, and had GO terms associated with the nucleus, protein binding, cytoplasm, ATP binding and regulation of transcription by RNA polymerase II. Among the 1,043 shared upregulated DE genes, more than 100 genes were associated with metabolic KEGG pathways, and many of them had GO terms related to the cytoplasm, cytosol and mitochondrion. Additionally, 34 genes were involved in pathways associated with neurodegeneration, including multiple diseases, such as 29 genes associated with Alzheimer’s disease, 28 genes associated with Huntington’s disease, and 26 genes associated with prion disease and Parkinson’s disease.

Finally, we determined which DE genes presented up- and downregulated expression conservation across both age groups and all timepoints (Supplementary material, Table S5). In total, 7 upregulated DE genes were found to be conserved : *G6PC3*,* IFI35*,* LOC122430223*,* IRF9*,* LGALS9*,* DHX58 and ISG15* as were 6 downregulated DE genes *STK26*, *EDEM3*, *ADSS2*, *CPNE3*,* STRN and ADAM10*.

### Ingenuity pathway analysis of differentially expressed genes between adult elk and calves

**Fig. 4:**
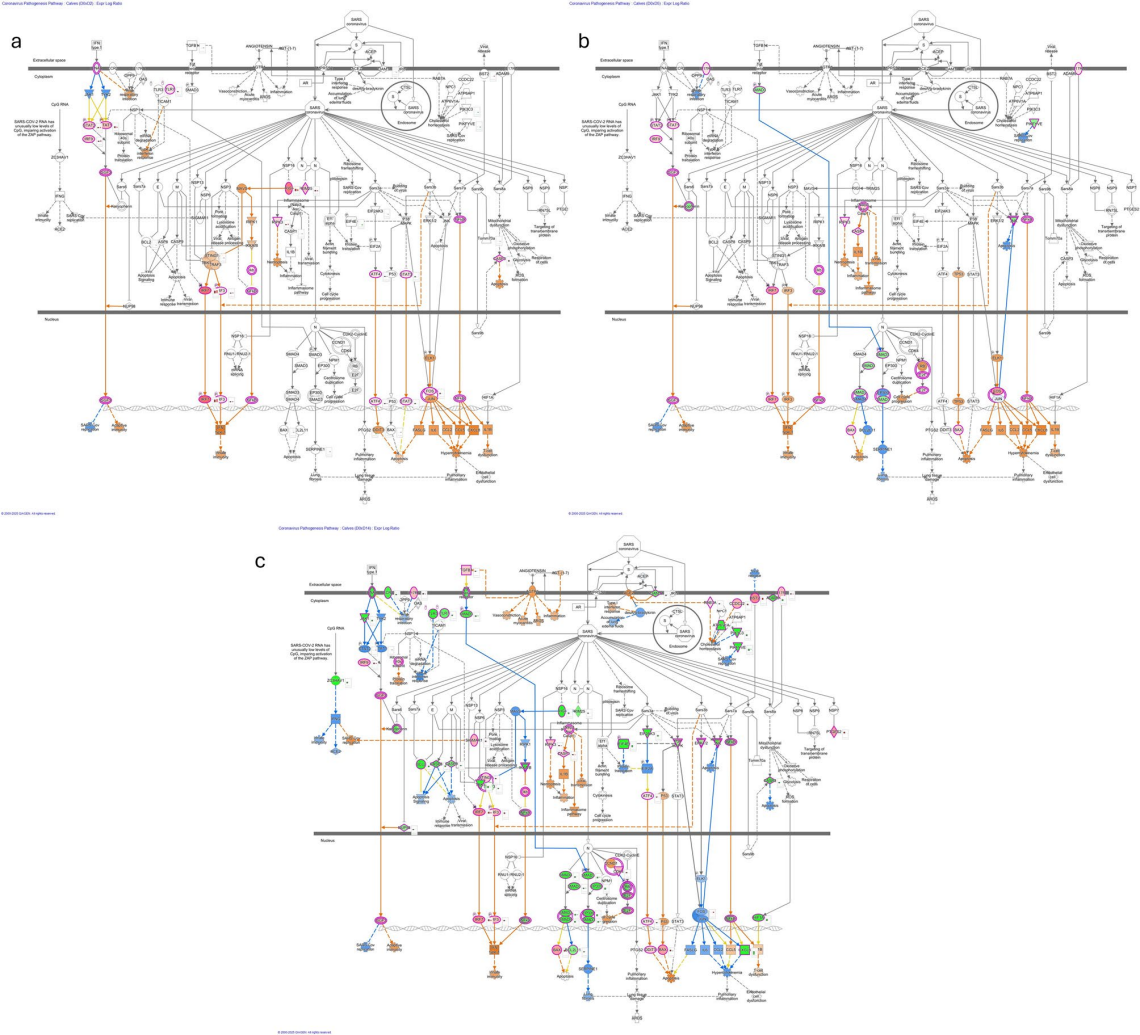
Ingenuity Pathway Analysis (IPA) showing the differentially expressed (DE) genes involved in the coronavirus pathogenesis pathway in elk calves. The predicted activated and inhibited pathway associations for the DE gene lists are shown for day 0 vs. day 2 (**a**), day 5 (**b**), and day 14 (**c**) post-SARS-CoV-2 infection. Downregulated genes are shown in green, and upregulated genes are shown in pink. The Molecule Activity Predictor tool was used to predict downstream activity on the basis of significant differential gene expression. Predicted activation is shown in orange, and predicted inhibition is shown in blue. The darker the fill is, the more confident the prediction. The solid lines represent direct relationships, whereas the dashed lines represent indirect relationships

**Fig. 5:**
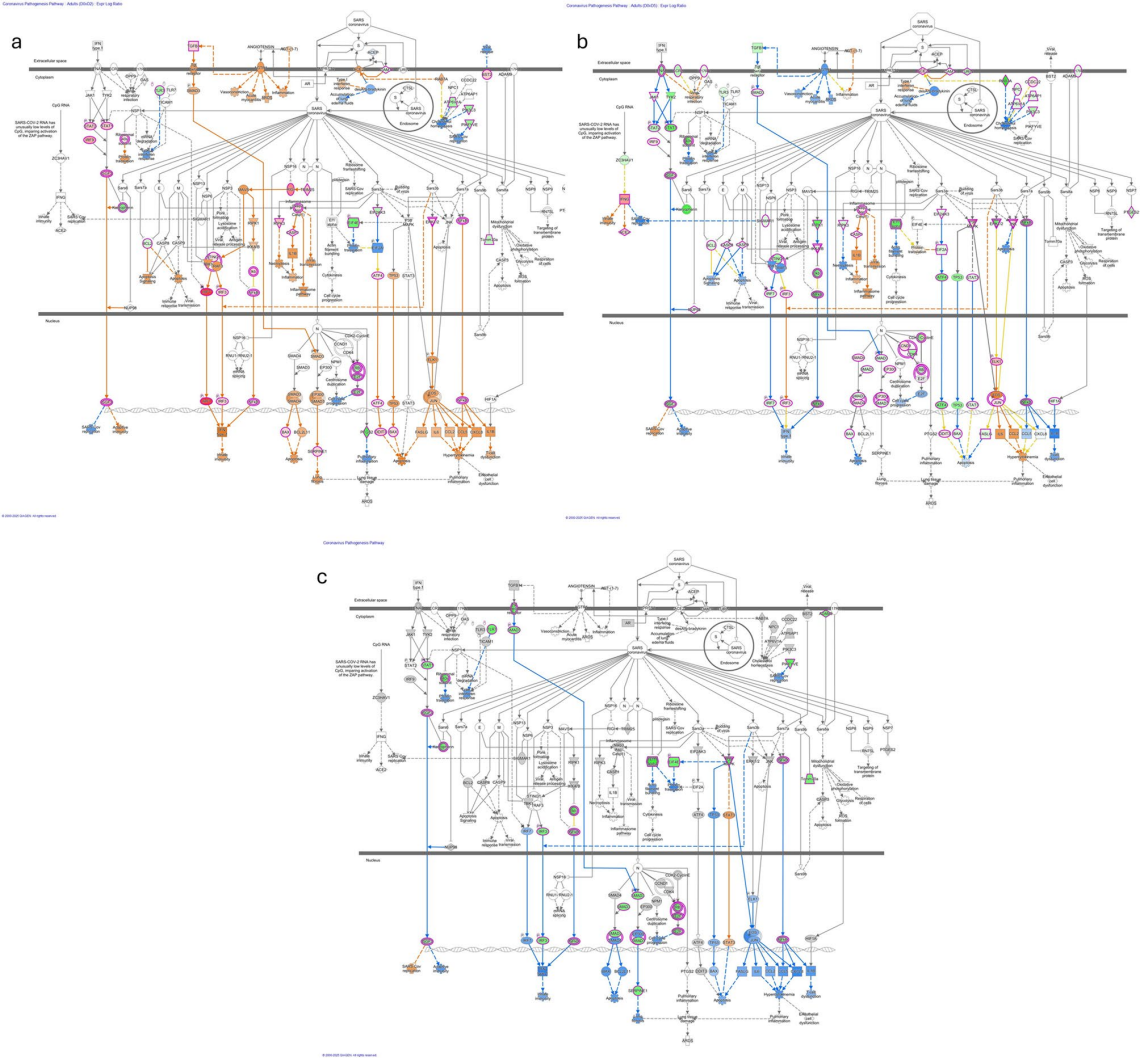
Ingenuity Pathway Analysis (IPA) showing the differentially expressed (DE) genes involved in the coronavirus pathogenesis pathway in adult elk. The predicted activated and inhibited pathway associations for the DE gene lists are shown for day 0 vs. day 2 (**a**), day 5 (**b**), and day 14 (**c**) post-SARS-CoV-2 infection. Downregulated genes are shown in green, and upregulated genes are shown in pink. The molecule activity predictor tool was used to predict downstream activity based on significant differential gene expression. Predicted activation is shown in orange, and predicted inhibition is shown in blue. The darker the fill is, the more confident the prediction. The solid lines represent direct relationships, whereas the dashed lines represent indirect relationships

To further analyze the elk response to SARS-CoV-2, we used the Ingenuity Pathway Analysis (IPA) software to examine changes in the coronavirus pathogenesis pathway. We compared the predicted intracellular responses at different stages of infection over time and between calves (Fig. 4) and adult elk (Fig. 5).

In calves, DE genes between day 2 pi and day 0 (Fig. 4a) suggest activation of the innate immune system. The molecular activity predictor tool highlighted the upregulation of the signal transducers and activators of transcription (STAT) pathway, with the *STAT1* and *STAT2* genes activated, which are known mediators of antiviral host defense and interferon response signaling. Similarly, the terms *IFN-1* and *ISGF3* were activated, contributing to proinflammatory NF-kB and interferon type 1 responses. Fig. 4b shows the comparison of DE genes from day 5 pi and day 0, where the *ISGF3* cascade remained activated in calves, along with *STAT1*, *STAT2*,* NF-kB* and Interferon type 1 responses. The IPA predicted the inhibition of SARS-CoV-2 replication and continued activation of the innate and adaptive immune responses, including associations with pathway terms such as apoptosis and cytokine storms by hypercytokinemia. A comparison of day 14 versus day 0 (Fig. 4c) revealed broad activation of pathway terms related to infection: *IFN-1* remained active, and SARS-CoV-2 replication was still predicted to be inhibited. Transforming growth factor (TGF)-beta receptor activity was inhibited, which was linked to the downregulation of the *SMAD3* gene. Notably, in calves, the spike protein receptor ACE2, which is necessary for virus entry into the cell, was predicted to be activated only on the day 14 DE gene list.

In adult elk, inflammatory signals associated with infection were more broadly activated in the IPA analysis of DE genes between day 2 pi and day 0 (Fig. 5a). The *TGFB*, *AGTR4 STAT1*, *STAT2*, and *ISGF3* genes were upregulated, along with Interferon type 1, adaptive, and innate immune activation. Interestingly, in contrast to calves, the *ACE2* receptor gene was predicted to be activated earlier in adult animals. A comparison of the DE gene predictions for day 5 pi and day 0 revealed a marked decrease in the immune response in adult animals (Fig. 5b). A ‘deactivation’ of Interferon type 1 response was seen, as well as a decrease in the involvement of *STAT1*, *STAT2*,* ISGF3*, *TGFB* and the *AGTR4* receptor. By day 5 pi in adults, the *ACE2* receptor and interferon type 1 response were still activated. However, in the DE gene lists from day 14 pi, the *ACE2* receptor gene was no longer activated, and the interferon type 1 response was inhibited along with a decrease in the expression level of other inflammatory genes, such as NF*-kB*, *STAT1* and *ISGF3* (Fig. 5c).

## Discussion

Here we characterize for the first time the changes in the peripheral blood gene expression profile of calves and adult elk experimentally challenged with SARS-CoV-2. Our data illustrates a broad and rapid immune response to SARS-CoV-2 inoculation. Gene expression profiles highlight the changing expression of genes related to important pathways for controlling infection and activating the host immune system including inflammatory and antiviral responses. These data offer an important resource for comparative studies in elk, other permissive cervid species such as white-tailed deer, and other mammals, including humans, to help understand the molecular machinery behind the antiviral response.

Although animals did not exhibit any clinical signs of infection, the differentially expressed gene analysis consistently revealed upregulated genes involved in SARS-CoV-2 infection processes and Coronavirus disease KEGG pathways in both calves and adult elk. In a recent publication from our group [8], we established that both elk calves and adults are susceptible to infection and have the ability to develop neutralizing antibodies against an ancestral SARS-CoV-2 variant (USA-WA1/2020). Notably, calves had a higher neutralizing response as well as displayed more inconsistent nasal and oral viral shedding compared to the adults possibly suggesting increased immune control of infection. While virus control is difficult to define, clearance of SARS-CoV-2 was not achieved as viral RNA and viral protein persisted in the retropharyngeal lymph nodes of both adults and calves.

Consistent with our previous work, results herein suggest that age may directly impact the elk response to SARS-CoV-2 challenge. The literature defines significant age-dependent differences in the immune response reported in humans [19, 20], where older individuals present more evidence of high morbidity and mortality than do younger individuals, who have a more effective immune response to spike and peptides against SARS-CoV-2. The severity of coronavirus disease (COVID-19) in adult humans could result from the decreased potential to immunomodulate excessive inflammation after strong cytokine secretion by T cells [21]. Consistent with those findings, in our study, the variation in the transcriptomic response between calves and adult elk was illustrated by the substantial increase in DE genes at all timepoints in the adult elk and the lack of conservation across DE genes from both ages. In the elk calves, immune activation and response trends were evident. Upregulated genes were associated with GO terms and KEGG pathways related to coronavirus disease by day 2 pi, but antiviral activation was emphasized for calves on day 5 pi, with a significant increase in the expression of genes related to an antiviral immune response, such as genes from the interferon regulatory factor (IFR) family, interferon gamma-inducible proteins, and other interferon-stimulated genes (ISGs). In calves by day 14 pi there was an increased number of DE upregulated genes associated with neurodegenerative disease KEGG pathways. While we did not observe any clinical signs associated with neurological disease in the calves, it is worth noting that in humans, neurological symptoms and neuroinflammatory events have been described during and following infection with SARS-CoV-2 [22, 23].

In adult elk, many DE genes were related to coronavirus disease - COVID-19 KEGG pathway and associated with immune system activation and the defense response to this coronavirus and other viral diseases. The GO and KEGG terms associated with COVID-19 appeared as early as day 2 pi, but by day 5 pi larger log2-fold changes in expression were observed. Some genes with larger log2-fold change are important to viral and bacterial pathogens infections including *VIM* (Vimentin) which has been shown to facilitate the binding of pathogens to the cell surface and promote their persistence in the host cells [24], and *TRIM15* which is in a class of antiviral proteins with components important for innate antiviral immunity [25]. Even 14 days pi coronavirus disease pathways were still significantly associated with DE genes in adults highlighting variation in immune responses by age, including the recognition and/or longevity of infection. For wildlife species especially, these results may suggest age variation in susceptibility and/or infection severity which has heavy implications for critical immune taxing events such as calving, and/or weaning. In turn this has implications for inter and intraspecies transmission events including those with domestic livestock.

Significantly DE genes conserved between calves and adults could serve as potential biomarkers of SARS-CoV-2 infection in wildlife and/or other hosts. Across calves and adult elk, seven upregulated DE genes were conserved across all time points: *G6PC3*,* IFI35*,* LOC122430223*,* IRF9*,* LGALS9*,* DHX58 and ISG15*. Glucose-6-phosphatase catalytic subunit 3 *(G6PC3)* is associated with macrophage activation, regulation of signal transduction, positive regulation of the defense immune response, and innate immune system activation [26]. Interferon-induced protein 35 (*IFI35*) is involved in the type I interferon signaling pathway and has been shown to be upregulated in patients with mild clinical COVID-19 [27]. Interferon regulatory factor 9 (*IRF9*) is an important component of the type I and III interferon signaling pathways, and is associated with *STAT1* and *STAT2*—also significantly DE genes in our study—to form the trimeric transcription factor *ISGF-3*, which is associated with cellular antiviral responses [28]. The *galectin-9* gene (*LGALS9*) is a beta-galactoside-binding protein that is involved in cell signaling, adhesion, and migration and is involved in inflammatory processes [29]. The LGALS9 protein has already been described as a potential biomarker for many diseases, including COVID-19, and is highly expressed in hospitalized patients [30]. Another study revealed that this gene potentially enhances virus replication and inflammation in epithelial cells in vitro, suggesting that galectin-9 impacts the early stage of SARS-CoV-2 infection [31]. The DExH-box helicase 58 (*DHX58*) gene is a member of a large family of RNA helicases and is responsible for encoding the LGP2 protein, which is important for RNA metabolism, splicing, recognition, modification, degradation, transport, and translation with antiviral properties [32]. The *ISG15* gene was upregulated at all post challenge timepoints, with high log2-fold changes early after inoculation (i.e., 3.90 at 2 days pi in calves and 4.94 at 2 days pi in adults). As an IFN-stimulated gene (ISG), *ISG15* is a key orchestrator of immune defense during viral infection and has already been described participating in the SARS-CoV-2 infection, inflammation, and immune responses [33]. An additional conserved gene that was DE in both calves and adults across all time points and has previously been reported in relation to SARS-CoV-2 infection was *TNFAIP3* (tumor necrosis factor, alpha-induced protein 3) where Sarlo-Davila et al. [] observed that the *TNFAIP3* gene was upregulated in deer respiratory epithelial cells *in vitro.*

It is known that some viruses can downregulate components of the immune system, including receptors necessary for host cell entry, to prevent reinfection of the same cell [34, 35]. Here, we also investigated the downregulated DE genes conserved between calves and adult elk at different timepoints and identified six genes: *STK26*, *EDEM3*, *ADSS2*, *ADAM10*, *CPNE3* and *STRN*. The *STK26* (Serine/Threonine Kinase 26), also known as MST4, is a protein kinase that is expressed in monocytes, T, B, and NK cells [36]. Compared with asymptomatic patients, the expression of *STK26* is increased in clinical human patients after SARS-CoV-2 infection [37]. A study investigating drug target proteins revealed that SARS-CoV-2 infection can change the structure of endoplasmic reticulum (ER) proteins at the initiation of virus‒cell interactions [38]. *EDEM3* (ER Degradation Enhancing Alpha-mannosidase Like Protein 3) is a member of a group of proteins that perform glycoprotein degradation in the ER [39]; *EDEM3* was downregulated in our study, indicating the possible interference of the virus in protein production. Adenylosuccinate synthase (ADSS) is known as a convertor of inosine monophosphate to adenosine monophosphate (AMP) and is present in vertebrates as two different isozymes: *ADSS1*, which is the basic form that participates in the purine nucleotide cycle, and the acidic form *ADSS2*, which catalyzes the synthesis of AMP [40, 41]. *Copine 3* (*CPNE3*) is a member of the CNPE family and expressed in the early stage of neutrophil differentiation [42], playing an important role in maintaining the normal function of pancreatic β-cells, regulating glucose uptake and insulin secretion [43]. *ADAM10* (ADAM metallopeptidase domain 10) is an adhesion receptor, and the mRNA that encodes this gene has been found in immune cells and is involved in the activation, differentiation and migration of T cells [44]. The *ADAM10* gene is also involved in the regulation of cytokine production and alternative protease cleavage, characterizing some important host dependency factors for SARS-CoV-2 entry into cells [45].

IPA illustrated the participation of DE genes in the activation of adaptive and innate immune responses during SARS-CoV-2 infection. As soon as day 2 pi, both ages of elk had signatures consistent with innate and adaptive immune activation as well as the inhibition of the replication of SARS-CoV-2, which is consistent with most host approaches to control infection. Specifically, interferon-associated terms such as Interferon Stimulated Factor 3 (*ISGF3)*, NF-kB, and general interferon type 1 responses were consistently activated. Type I interferons and associated mediators are known to play protective roles in controlling viral infections [46]. This includes *IRF3* (interferon regulatory factor 3), the primary early regulatory factor responsible for inducing IFN-1 during viral infection, which positively regulates apoptosis and inhibits NF-kB translocation [46, 47]. *IRF7* (interferon regulatory factor 7) can mediate inflammatory responses, and a lack of *IRF9* results in increased COVID-19 risk [46]. Broadly, the host immune response, inflammatory, and antiviral signaling pathways for the adult elk peak in the day 2 pi profile, but appeared to decline over time, as evidenced by the inhibition of immune terms by day 5 pi. In contrast, the calves appeared to show an increase in the immune response and interferon activation through day 5 pi and only moderately decreased in activity by day 14 di. Notably, adults showed a predicted involvement of *ACE2* as early as day 2 and through day 5 pi, whereas calves showed activation of *ACE2* only on day 14 pi.

Both calves and adult elk have been shown to lack clinical signs associated with SARS-CoV-2 , suggesting that while elk can become infected, it is successful at controlling the infection without causing disease. This contrasts with white-tailed deer, which appear more permissive to infection, and emphasizes the importance of investigating species-specific immune responses in cervids. While the restriction of viral RNA to the medial retropharyngeal lymph nodes of elk may have been a result of the mode of intranasal inoculation, intranasal inoculation of white-tailed deer infected in an identical fashion exhibited widespread tissue tropism of SARS-CoV-2. This work provides unique insight into potential mechanisms of viral control by the host immune system, variation in disease responses among age groups, and gene signature associations with stages of infection which are largely under-investigated in elk and other ungulates. While the present study captures insights into the systemic peripheral host immune response to SARS-CoV-2 infection, we recognize work characterizing additional timepoints, animal to animal variation, immune tissues, and localized responses to experimental challenge would be of further value.

## Conclusions

Here, for the first time, we describe the peripheral transcriptional response of North American elk to SARS-CoV-2 infection at different timepoints and in different age groups. Overall, this work facilitates the discussion of species-specific responses to a zoonotic pathogen with global human health implications, provides further characterization of wildlife immune responses with an emphasis on cervid investigation, and highlights the gene expression profiles associated with SARS-CoV-2.

## Methods

### Elk challenge and sample collection

 All procedures and animal work were performed following approval by the NADC Animal and Care Use Committee (IACUC) and following the Guide for Care and Use of Laboratory Animals regulations. For the challenge studies, adult elk cows from the sustained research herd at the NADC campus (*n* = 10), approximately 4 years of age, were transferred to agricultural biosafety level 3 (AgBSL3) facilities and allowed to acclimate for 2 weeks. Elk calves (*n* = 11, mixed sex), approximately 5 months of age, were born in high containment, and remained with dams until weaning. All animals were challenged with an ancestral strain of SARS-CoV-2 (USA-WA1/2020), as described previously in Boggiatto et al. [7]. Briefly, the animals were sedated and challenged intranasally with 5 mL total volume of a SARS-CoV-2 isolate (USA-WA1/2020 – SARS-Related Coronavirus 2, Isolates hCoV-19/USA-WA1/2020) using a mucosal atomization device (MAD). Virus was sourced from BEI Resources stock (NR-52281), passaged three times in Vero E6 cells, and titered to a challenge dose of 10^6^ TCID50/mL for adults and 10^5.5^ TCID50/mL for elk calves.

 Blood samples were collected from all elk calves and elk adult cows pre-challenge day 0 (control), day 2, day 5, and day 14 post- inoculation using PAXgene^®^ Blood RNA tubes (BD Biosciences, San Jose, CA). On days 2 and 5 post infection two adult and two calves were euthanized for pathology and immunohistochemistry analysis. PAX tubes were stored at −8°C until subsequent extraction of all samples.

### RNA isolation, library preparation and sequencing

 Total RNA was isolated using MagMAX™ for Stabilized Blood Tubes RNA Isolation Kit, which are compatible with PAXgene™ Blood RNA Tubes (catalog number 4451894) (Thermo Fisher Scientific, Waltham, MA). The RNA integrity was determined via a bioanalyzer, and the average RNA integrity number (RIN) for all the samples was 9.3. Library preprocessing was performed via an Illumina mRNA kit, and sequencing was performed at the University of Illinois, Urbana-Champaign, at Roy J. Carver Biotechnology Center via Nova Seq X Plus, which generated 150 base pair paired-end reads.

### RNA sequencing bioinformatics: quality control and read mapping

 Data processing was performed using the Nextflow (version 24.04.2) nf-core/rna-seq pipeline (version 3.14.0)[48,49]. Read quality control (QC) was processed by FastQC software (https://www.bioinformatics.babraham.ac.uk/projects/fastqc/,version0.12.1), and the library adaptors were trimmed using TRIMgalore software (https://www.bioinformatics.babraham.ac.uk/projects/trim_galore/, version 0.6.7), removing the reads with phred quality scores lower than 20. After filtering, samples retained between 40M and 53M high-quality mapped reads (MQ>0), for adult elk and between 41M and 60M for calves, summarized by MultiQC report ([50], version 1.15). Sequence reads were aligned against the *Cervus canadensis *reference genome (NCBI assembly: ASM19320065v1) via STAR software ([51], version 2.7.10a). Gene counts were performed with featureCounts software ([52], release 2.0.4), and all downstream statistical analyses were performed in R.

### Differentially expressed gene analysis

The differentially expressed (DE) analysis was performed comparing each challenged day post-inoculation (day 2, day 5 and day 14) to the control group (day 0) for both the adult and calf groups, using DESeq2 software in R [53], version 1.44.0. For the DE analysis, reads were filtered to remove genes with no expression (zero reads), genes with very low expression (less than 1 read per sample on average), and genes with rare expression across the samples (genes with read counts that were not present in at least 3 samples).

### GO term enrichment analysis and ingenuity pathway analysis

Gene Ontology (GO) enrichment analysis and KEGG pathway analysis were performed using DAVID bioinformatics software (https://david.ncifcrf.gov/tools.jsp) to help interpret and summarize the biological processes and pathways associated with the DE genes. The conservation between DE genes in the different groups was determined via Venny ([54], version 2.1]). Ingenuity pathway analysis (IPA) (QIAGEN, Redwood City, CA, USA) [55] was performed using the gene expression data as input to predict the activation or inhibition of the metabolic canonical pathways in a core analysis.

## Supplementary Information

Supplementary Material 1: Table S1. List of upregulated and downregulated differentially expressed genes in elk calves 2, 5 and 14 days after SARS-CoV-2 challenge

Supplementary Material 2: Table S2. List of GO terms and KEGG pathways associated with up- and downregulated differentially expressed genes in elk calves 2, 5 and 14 days after SARS-CoV-2 challenge

Supplementary Material 3: Figure S1. Functional enrichment analysis of up- and downregulated genes in calves on day 2 post-infection. Dot plots summarize KEGG pathways and Gene Ontology (GO) term enrichment analysis for differentially expressed genes. Each panel presents the top 20 significantly enriched terms based on *p*-value ranking. Shown are KEGG pathway enrichment terms for – upregulated (a) and downregulated (b) genes. GO term enrichment terms are also shown for upregulated (c) and downregulated genes (d). Dot sizes indicated the number of genes associated with each pathway (count), and dot colors represent statistical significance (-log10(*p*-value)).

Supplementary Material 4: Figure S2. Functional enrichment analysis of up- and downregulated genes in calves on day 5 post-infection. Dot plots summarize KEGG pathways and Gene Ontology (GO) term enrichment analysis for differentially expressed genes. Each panel presents the top 20 significantly enriched terms based on *p*-value ranking. Shown are KEGG pathway enrichment terms for – upregulated (a) and downregulated (b) genes. GO term enrichment terms are also shown for upregulated (c) and downregulated genes (d). Dot sizes indicated the number of genes associated with each pathway (count), and dot colors represent statistical significance (-log10(*p*-value)).

Supplementary Material 5: Figure S3. Functional enrichment analysis of up- and downregulated genes in calves on day 14 post-infection. Dot plots summarize KEGG pathways and Gene Ontology (GO) term enrichment analysis for differentially expressed genes. Each panel presents the top 20 significantly enriched terms based on *p*-value ranking. Shown are KEGG pathway enrichment terms for – upregulated (a) and downregulated (b) genes. GO term enrichment terms are also shown for upregulated (c) and downregulated genes (d). Dot sizes indicated the number of genes associated with each pathway (count), and dot colors represent statistical significance (-log10(*p*-value)).

Supplementary Material 6: Table S3. List of up- and downregulated differentially expressed genes in adult elk 2, 5 and 14 days after SARS-CoV-2 challenge

Supplementary Material 7: Table S4. List of GO terms and KEGG pathways associated with up- and downregulated genes in adult elk 2, 5 and 14 days after SARS-CoV-2 challenge

Supplementary Material 8: Figure S4. Functional enrichment analysis of up- and downregulated genes in adult elk on day 2 post-infection. Dot plots summarize KEGG pathways and Gene Ontology (GO) term enrichment analysis for differentially expressed genes. Each panel presents the top 20 significantly enriched terms based on *p*-value ranking. Shown are KEGG pathway enrichment terms for– upregulated (a) and downregulated (b) genes. GO term enrichment terms are also shown for upregulated (c) and downregulated genes (d). Dot sizes indicated the number of genes associated with each pathway (count), and dot colors represent statistical significance (-log10(*p*-value)).

Supplementary Material 9: Figure S5. Functional enrichment analysis of up- and downregulated genes in adult elk on day 5 post-infection. Dot plots summarize KEGG pathways and Gene Ontology (GO) term enrichment analysis for differentially expressed genes. Each panel presents the top 20 significantly enriched terms based on *p*-value ranking. Shown are KEGG pathway enrichment terms for– upregulated (a) and downregulated (b) genes. GO term enrichment terms are also shown for upregulated (c) and downregulated genes (d). Dot sizes indicated the number of genes associated with each pathway (count), and dot colors represent statistical significance (-log10(*p*-value)).

Supplementary Material 10: Figure S6. Functional enrichment analysis of up- and downregulated genes in adult elk on day 14 post-infection. Dot plots summarize KEGG pathways and Gene Ontology (GO) term enrichment analysis for differentially expressed genes. Each panel presents the top 20 significantly enriched terms based on *p*-value ranking. Shown are KEGG pathway enrichment terms for– upregulated (a) and downregulated (b) genes. GO term enrichment terms are also shown for upregulated (c) and downregulated genes (d). Dot sizes indicated the number of genes associated with each pathway (count), and dot colors represent statistical significance (-log10(*p*-value)).

Supplementary Material 11: Table S5. List of upregulated and downregulated differentially expressed genes conserved across adult elk and calves for all timepoints. 

## Data Availability

The dataset supporting the conclusions of this article is available in the SRA-NCBI repository under project number PRJNA1196870 (https://www.ncbi.nlm.nih.gov/bioproject/?term=PRJNA1196870).
