# Supplementary figures and images for: Peripheral transcriptional responses to experimental SARS-CoV-2 inoculation in North American elk cows and calves
Source: BMC Genomics. 2025 Aug 28;26:781. doi: 10.1186/s12864-025-11956-5 (PMC12392485; doi:10.1186/s12864-025-11956-5)

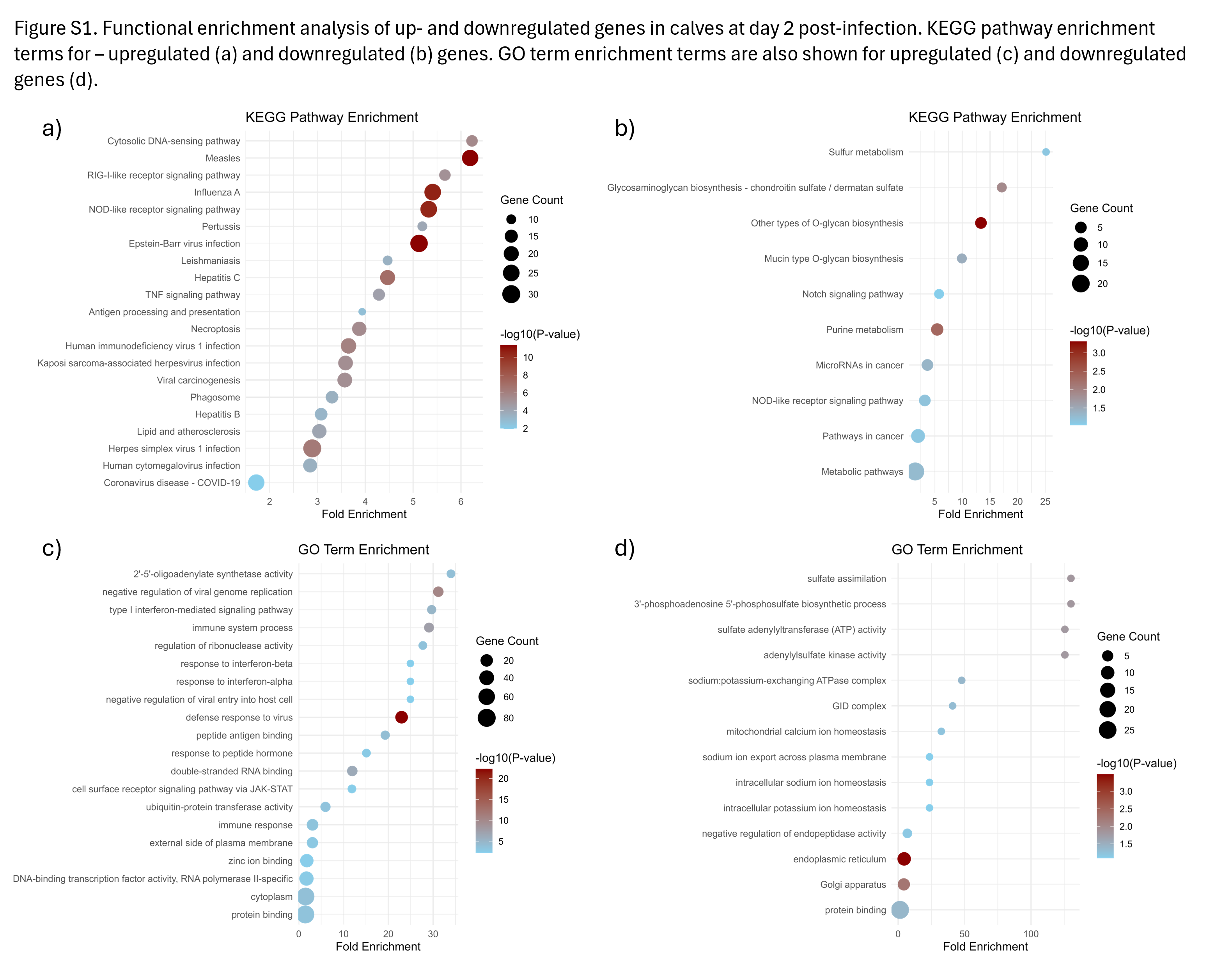

Supplement: Supplementary file 3 — Supplementary Material 3: Figure S1. Functional enrichment analysis of up- and downregulated genes in calves on day 2 post-infection. Dot plots summarize KEGG pathways and Gene Ontology (GO) term enrichment analysis for differentially expressed genes. Each panel presents the top 20 significantly enriched terms based on p-value ranking. Shown are KEGG pathway enrichment terms for – upregulated (a) and downregulated (b) genes. GO term enrichment terms are also shown for upregulated (c) and downregulated genes (d). Dot sizes indicated the number of genes associated with each pathway (count), and dot colors represent statistical significance (-log10(p-value)). [file 12864_2025_11956_MOESM3_ESM.png]

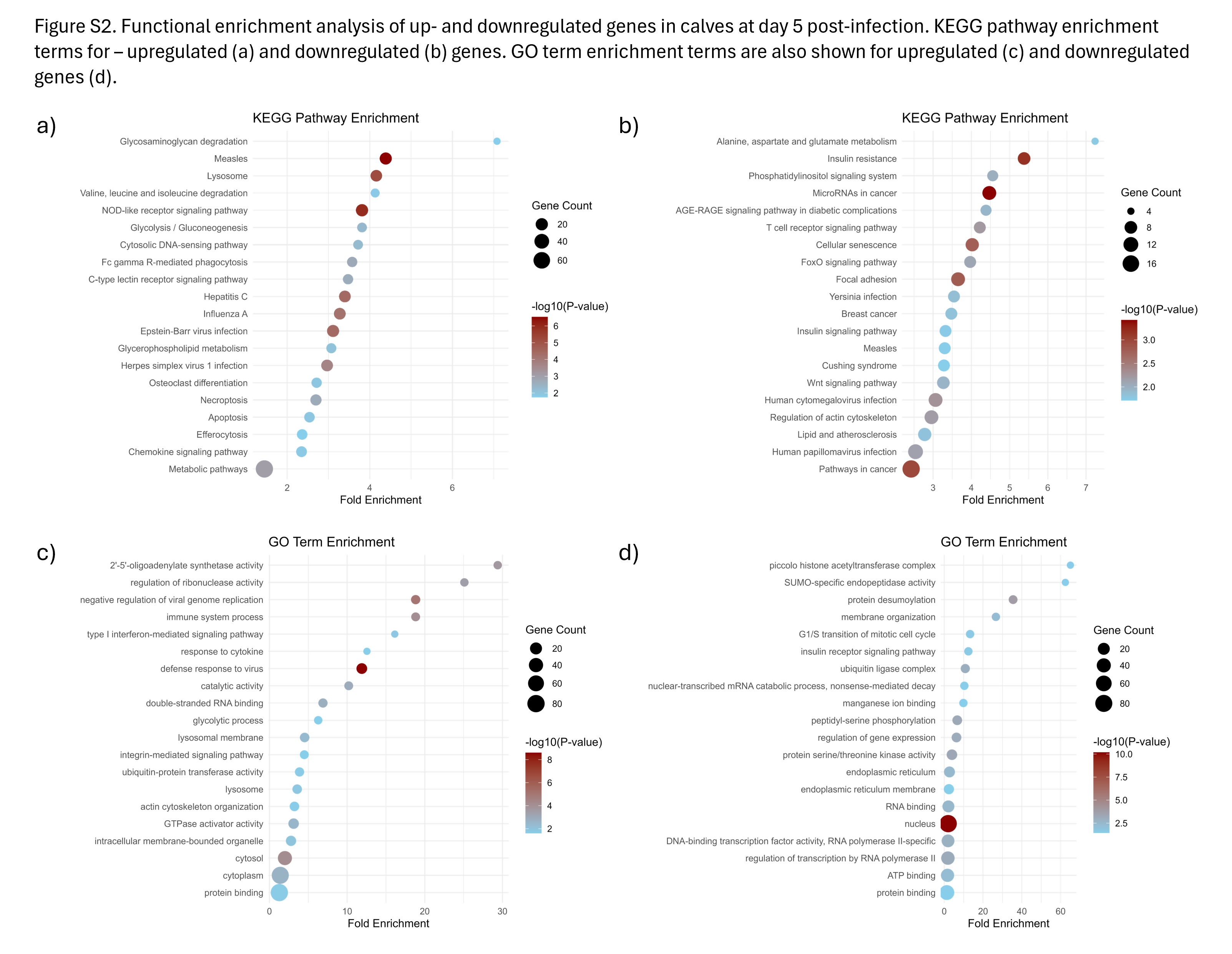

Supplement: Supplementary file 4 — Supplementary Material 4: Figure S2. Functional enrichment analysis of up- and downregulated genes in calves on day 5 post-infection. Dot plots summarize KEGG pathways and Gene Ontology (GO) term enrichment analysis for differentially expressed genes. Each panel presents the top 20 significantly enriched terms based on p-value ranking. Shown are KEGG pathway enrichment terms for – upregulated (a) and downregulated (b) genes. GO term enrichment terms are also shown for upregulated (c) and downregulated genes (d). Dot sizes indicated the number of genes associated with each pathway (count), and dot colors represent statistical significance (-log10(p-value)). [file 12864_2025_11956_MOESM4_ESM.png]

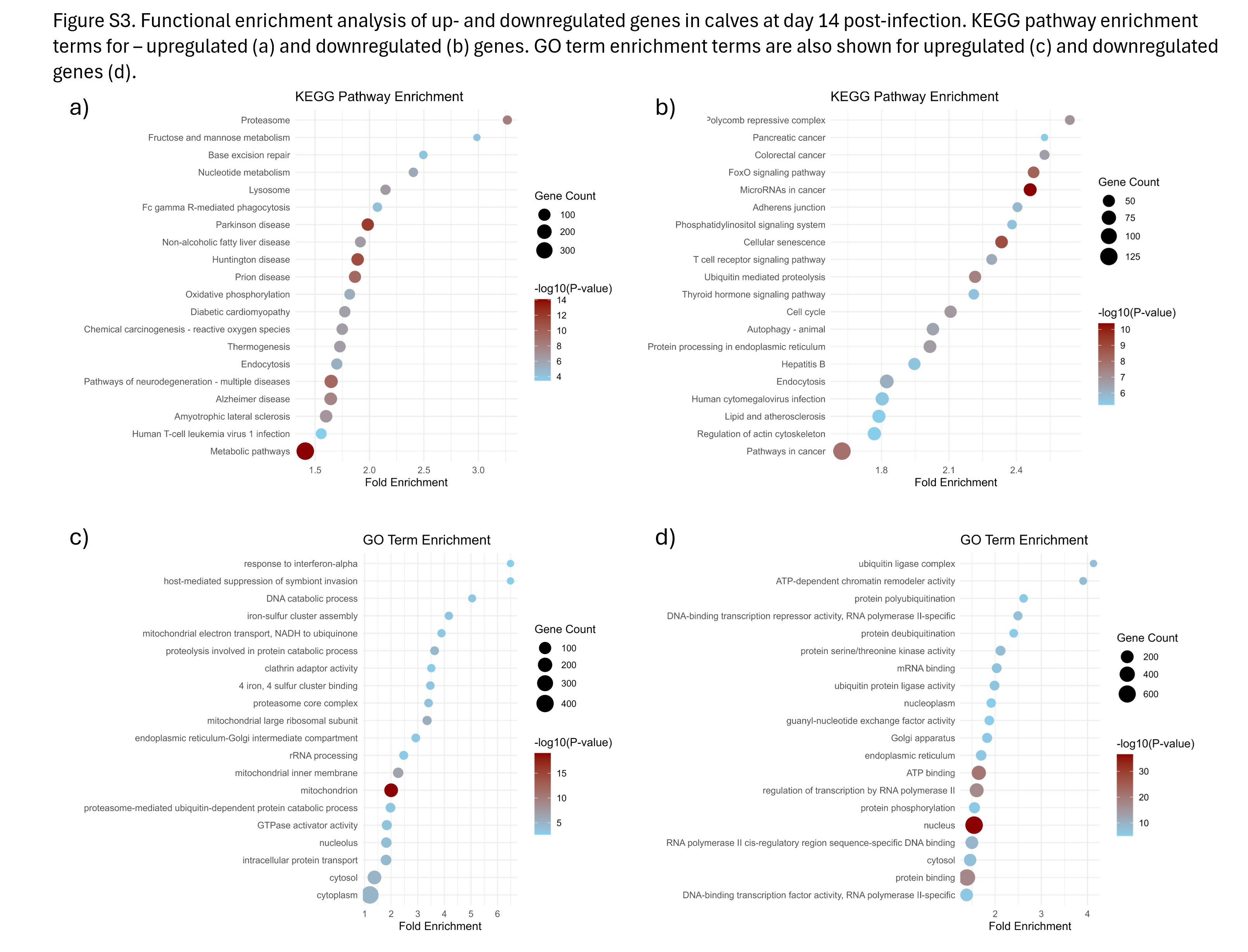

Supplement: Supplementary file 5 — Supplementary Material 5: Figure S3. Functional enrichment analysis of up- and downregulated genes in calves on day 14 post-infection. Dot plots summarize KEGG pathways and Gene Ontology (GO) term enrichment analysis for differentially expressed genes. Each panel presents the top 20 significantly enriched terms based on p-value ranking. Shown are KEGG pathway enrichment terms for – upregulated (a) and downregulated (b) genes. GO term enrichment terms are also shown for upregulated (c) and downregulated genes (d). Dot sizes indicated the number of genes associated with each pathway (count), and dot colors represent statistical significance (-log10(p-value)). [file 12864_2025_11956_MOESM5_ESM.png]

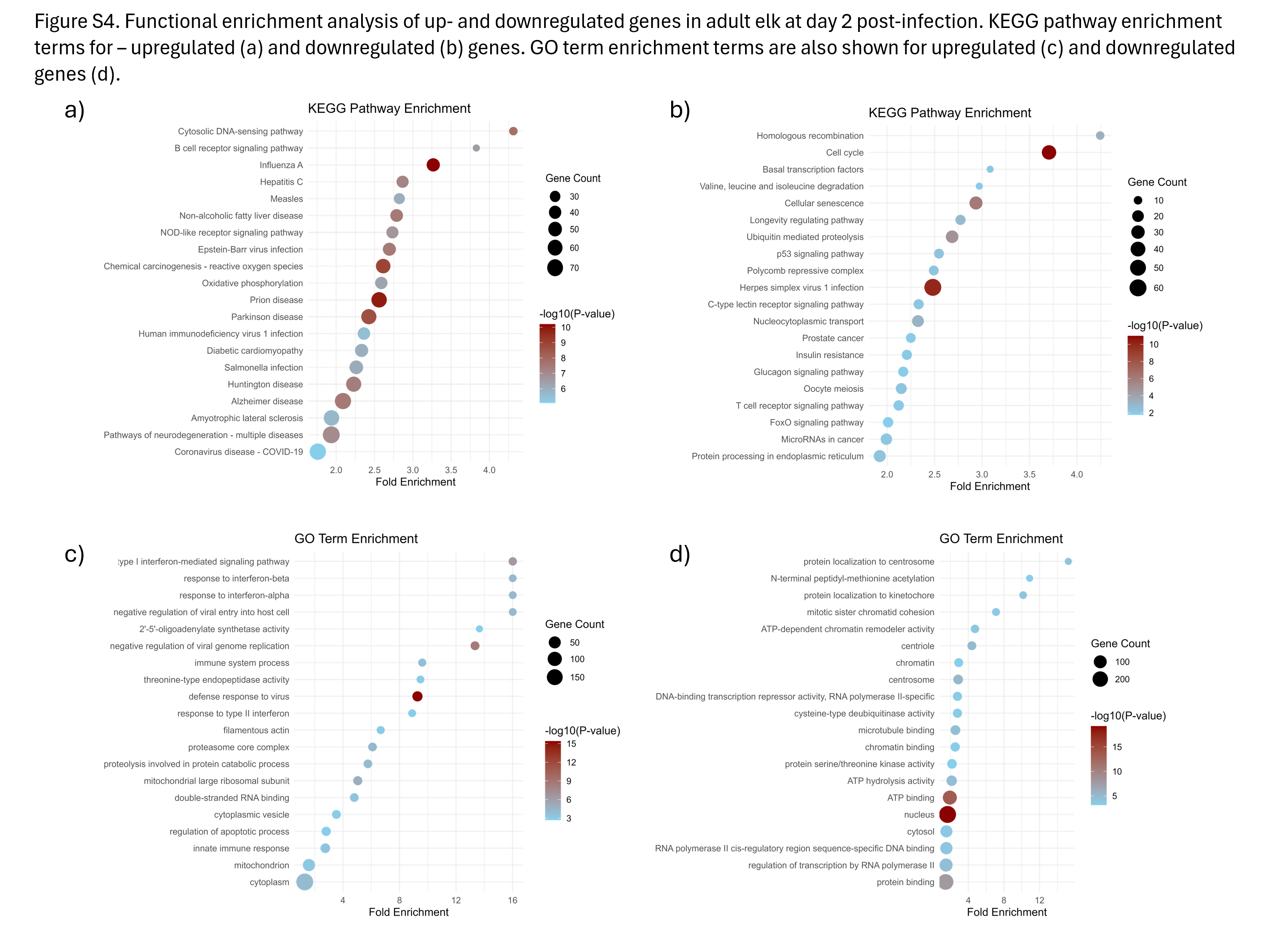

Supplement: Supplementary file 8 — Supplementary Material 8: Figure S4. Functional enrichment analysis of up- and downregulated genes in adult elk on day 2 post-infection. Dot plots summarize KEGG pathways and Gene Ontology (GO) term enrichment analysis for differentially expressed genes. Each panel presents the top 20 significantly enriched terms based on p-value ranking. Shown are KEGG pathway enrichment terms for– upregulated (a) and downregulated (b) genes. GO term enrichment terms are also shown for upregulated (c) and downregulated genes (d). Dot sizes indicated the number of genes associated with each pathway (count), and dot colors represent statistical significance (-log10(p-value)). [file 12864_2025_11956_MOESM8_ESM.png]

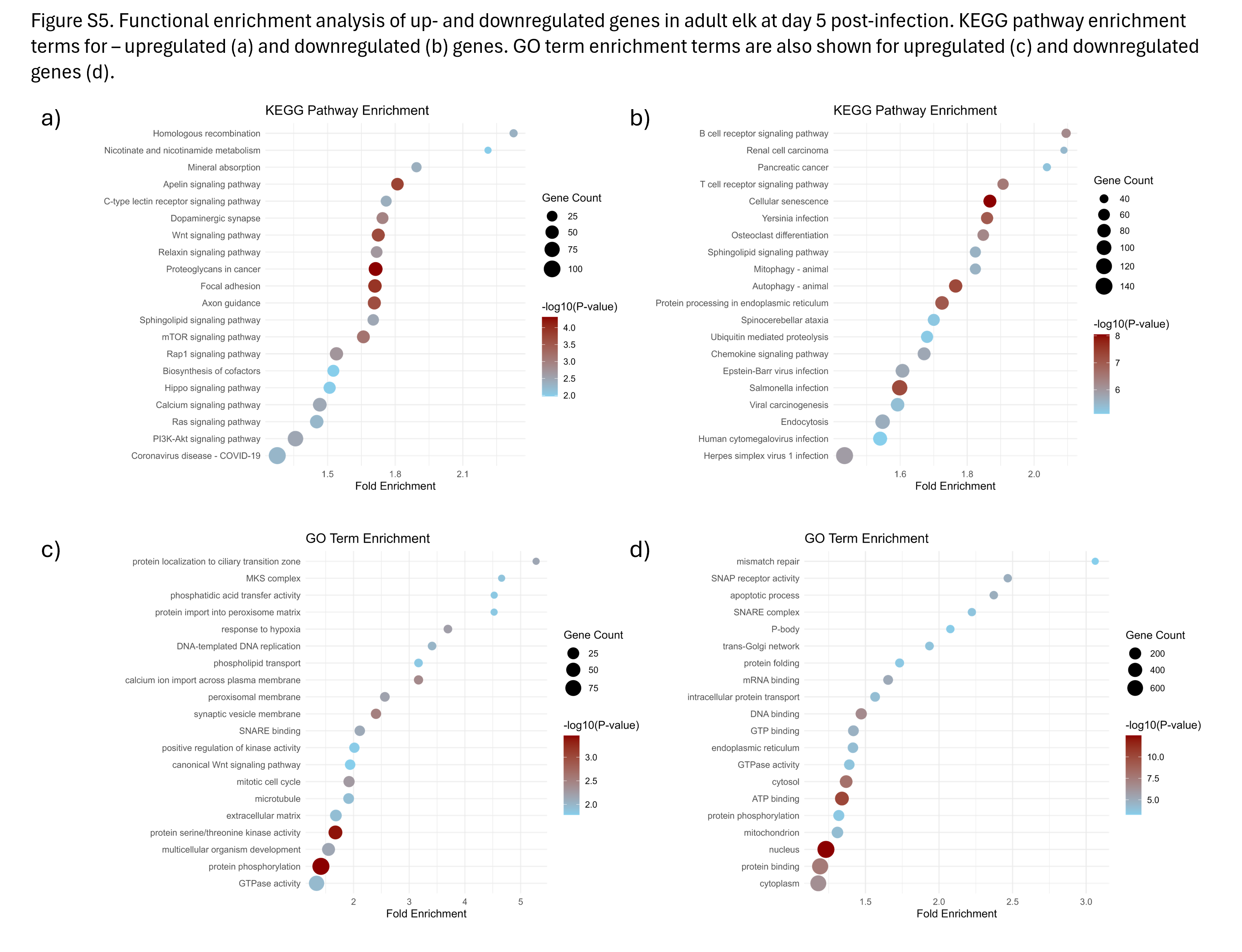

Supplement: Supplementary file 9 — Supplementary Material 9: Figure S5. Functional enrichment analysis of up- and downregulated genes in adult elk on day 5 post-infection. Dot plots summarize KEGG pathways and Gene Ontology (GO) term enrichment analysis for differentially expressed genes. Each panel presents the top 20 significantly enriched terms based on p-value ranking. Shown are KEGG pathway enrichment terms for– upregulated (a) and downregulated (b) genes. GO term enrichment terms are also shown for upregulated (c) and downregulated genes (d). Dot sizes indicated the number of genes associated with each pathway (count), and dot colors represent statistical significance (-log10(p-value)). [file 12864_2025_11956_MOESM9_ESM.png]

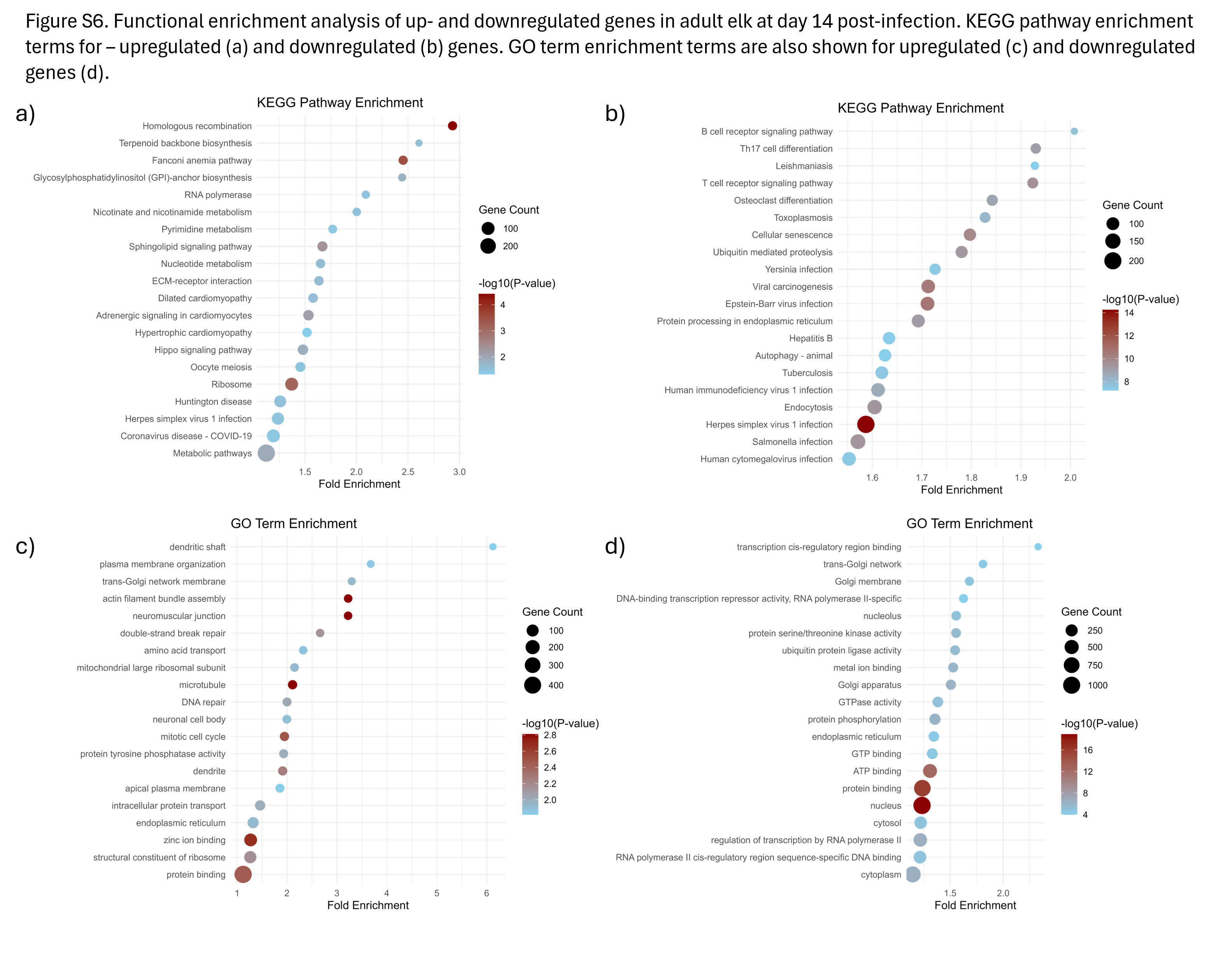

Supplement: Supplementary file 10 — Supplementary Material 10: Figure S6. Functional enrichment analysis of up- and downregulated genes in adult elk on day 14 post-infection. Dot plots summarize KEGG pathways and Gene Ontology (GO) term enrichment analysis for differentially expressed genes. Each panel presents the top 20 significantly enriched terms based on p-value ranking. Shown are KEGG pathway enrichment terms for– upregulated (a) and downregulated (b) genes. GO term enrichment terms are also shown for upregulated (c) and downregulated genes (d). Dot sizes indicated the number of genes associated with each pathway (count), and dot colors represent statistical significance (-log10(p-value)). [file 12864_2025_11956_MOESM10_ESM.png]
